# Effect of Cannabinoid Receptor Activation on Spreading Depression

**Published:** 2012

**Authors:** Hadi Kazemi, Mehdi Rahgozar, Erwin-Josef Speckmann, Ali Gorji

**Affiliations:** 1 *Shefa Neuroscience Research Centre, Tehran, Iran*; 2 *Department of Pediatrics, Shahed University, Tehran, Iran *; 3 *Institut für Physiologie I, Westfalische Wilhelms-Universitat Munster, Münster, Germany*; 4 *Institut für Experimentelle Epilepsieforschung, Westfalische Wilhelms-Universitat Munster, Münster, Germany*

**Keywords:** Endocannabinoid, Headache, Marijuana, Migraine, Pharmacotherapy, Synaptic transmission

## Abstract

**Objective(s):**The objective of this study was to evaluate the effect of cannabinoid on cortical spreading depression (CSD) in rat brain. Cannabis has been used for centuries for both symptomatic and prophylactic treatment of different types of headaches including migraine. CSD is believed to be a putative neuronal mechanism underlying migraine aura and subsequent pain.

**Materials and Methods:**The effects of Delta9-tetrahydrocannabinol (THC), as well as, cannabinoid CB1 and CB2 receptor agonists on CSD in rat neocortical slices were investigated. Furthermore, the effect of cannabinoid CB1 agonist was tested on field excitatory postsynaptic potentials (fEPSP) and long-term potentiation (LTP).

**Results:**HC (1-20 microM) dose dependently suppressed CSD amplitude, duration, and propagation velocity. Cannabinoid CB1 agonist, WIN 55,212-2 mesylate (1-10 microM), also significantly suppressed all characteristic features of CSD. However, cannabinoid CB2 agonist, JWH-133 (1-20 microM), did not affect CSD. FEPSP and induction of LTP were suppressed by application of WIN55212-2.

**Conclusion:**Suppression of CSD by activation of CB1 receptors points to the potential therapeutic effects of cannabinoids in migraine with aura. More research is needed before we know whether cannabinoids may be helpful in treating migraine pain.

## Introduction

Cannabis has been traditionally used as a therapeutic agent in central nervous system disorders such as epilepsy and migraine headache for several centuries (1, 2). A large number of studies using Delta9-tetrahydrocannabinol (THC), the main pharmacologically active constituent of cannabis, or cannabinoid synthetic derivatives have substantially contributed to advance the understanding of the neurobiological mechanisms produced by cannabinoid receptor activation (1). 

Cannabinoids evoke various central effects, such as sedation and analgesia via an activation of the CB1 (3, 4). It has been suggested that cannabinoid receptors may play a significant role in modulation of nociception as well as in psychomotor control, memory function, neuroendocrine regulation, control of movement, appetite regulation, emesis, and many other brain functions (5). 

Migraine has numerous relationships to endocannabinoid functions. Endocannabinoid deficiency has been suggested to underlie the pathophysiology of migraine (1). The attributes of cannabis to affect serotonergic, dopaminergic, anti-inflammatory and glutamate mechanisms of migraine have rendered it a proposed drug for headache treatment, although clinical studies providing a scientific basis for the potential efficacy of cannabinoids in migraine are limited. Several studies revealed a potent cannabinoid agonistic activity at 5-HT1A receptors and an antagonistic property at 5-HT2A receptors, which suggest the putative efficacy of therapeutic cannabinoids in acute migraine and in its prophylactic treatment, respectively (1, 3, 6). The midbrain periaqueductal grey matter, a putative migraine generator area, was shown to be modulated by endocannabinoids (7). Therapeutic potentials of cannabinoid receptors in migraine was suggested by the observations that cannabinoids inhibited neuronal firing in the trigeminocervical complex, neurogenic dural vasodilatation, and calcitonin gene-relatad peptide (CGRP)-, capsaicin-, and nitric oxide (NO)-induced dural vessel dilation induced by trigeminovascular stimulation (8, 9). The preclinical data supporting the antinociceptive role of cannabinoids, and some clinical data noting their benefit in pain, indicate that further research is needed before cannabinoids are recommended clinically for pain or headache (10).

Cortical spreading depression (CSD) refers to a phenomenon that manifests as a self-propagating wave of neuronal hyperexcitability followed by a transient depression (11, 12). CSD is accompanied by characteristic ionic, metabolic, and hemodynamic changes and may play an essential role in some neurological disorders including migraine with aura (13, 14). The hypothesis that the aura is the human equivalent of CSD has been well established (15). Propagation of a CSD-like wave in human neocortical tissues generates aura symptoms in migrainous patients (16). Furthermore, it was proposed that CSD might also trigger the rest of the migraine attacks including pain (17-20). To investigate the possible actions of cannabinoids on CSD, the present study was designed to determine the effect of THC as well as CB1 and CB2 agonists on different characteristic features of CSD in rat neocortical tissues.

## Materials and Methods

The experiments were performed on adult rat (250-300 g) somatosensory neocortical slices. The brain was removed under deep methohexital anaesthesia and placed in cold (1–4 °C) artificial cerebrospinal fluid (ACSF) pre-equilibrated with 5% CO_2_ in O_2_ to give a pH of 7.4. The ACSF contained (in mM): NaCl 124, KCl 4, CaCl_2_ 1.0, NaH_2_PO_4_ 1.24, MgSO_4_ 1.3, NaHCO_3_ 26 and glucose 10. The somatosensory neocortices were dissected and cut into slices of 500 µm thickness. The slices were incubated in ACSF solution for >1 hr at 28 °C. After 30 min of incubation, CaCl_2_ was elevated to 2.0 mM. Slices were transferred to an interphase-type experimental chamber and superfused with ACSF at 32 °C (1.5–2 ml/min). 

***Electrophysiological recordings***

Extracellular field potentials were recorded with glass microelectrodes (150 mM/l NaCl; 2–10 MΩ) connected to the amplifier by an Ag/AgCl–KCl bridge in the third and the fifth layers of neocortical tissues. Traces were digitized by *Digidata* 1200 (Axon Instruments, CA, ) and the data were collected and analyzed by *Axoscope* 10 (Axon Instruments, CA, ). 

***Induction of CSD***

CSD was elicited by KCl microinjection. A glass electrode filled with 2 M KCl was fixed in a special holder connected with plastic tube to a pressure injector and the tip inserted into the layer I-II of the neocortical slices. A high-pressure pulse was applied to inject an amount of K^+^ in the tissue sufficient to induce CSD (tip diameter: 2 µm; injection pressure 0.5–1.0 bar applied for 200–300 ms, two injections, 1–3 nl per pulse). CSD-like events were evaluated with respect to their amplitude, duration and velocity rates. CSD duration was defined as the interval between the time of half-maximal voltage shift during onset and recovery of the negative DC potential deflection (21, 22).

***Long-term potentiation***

Single pulses of electrical stimulation were applied through a bipolar platinum electrode attached to the white matter perpendicular to the recording electrodes. Evoked field excitatory postsynaptic potentials (fEPSP) were recorded in the third layer of neocortical slices. The fEPSP was elicited by adjusting the intensity of stimulation to ~50% of that at which population spikes after fEPSP began to appear. The amplitude of fEPSP 1 ms after the onset was measured for data analysis. In long-term potentiation (LTP) experiments, the cortex was sequentially stimulated once every minute. Ten trains of four pulses at 100 Hz were delivered 200 ms to the white matter of neocortical slices. LTP was operationally defined as the mean change in fEPSP amplitude in response to five stimuli given 30 min after tetanic stimulation compared with the mean response to five test pulses applied immediately before the stimulation. Thus % potentiation= [(post-tetanus amplitude of fEPSP/baseline amplitude of fEPSP) 1] 100. Tetanic stimulation was applied 60 min after application of drug (21, 22). 

***Experimental protocols***

Two different experimental protocols were used, each of which consisted of several periods.

The first experimental protocol consisted of four periods as follows: (a) control period, neocortical slices were superfused with ACSF (30 min), tested for spontaneous CSD; (b) KCl injection, induction of CSD (CSD1); (c) application of THC (0.1-20 µM), the CB1 agonist WIN 55,212-2 mesylate (0.1-10 µM), or the CB2 agonist JWH 133 (1-20 µM, 60 min) before the second injection of KCl (CSD2); (d) washout of THC, WIN 55,212-2 mesylate, or JWH 133 with ASCF (45 min, second control period), third injection of KCl (CSD3). Only a single concentration of THC, WIN 55,212-2 mesylate, or JWH 133 was used in a given slice. In control experiments, DMSO (0.5%) was added to the bath solution after the first KCl injection (60 min) and washed with ASCF (45 min) after the second and before the third KCl application.

The second experimental protocol consisted of four periods as follows: (a) control period, neocortical slices were superfused with ACSF (30 min), tested for spontaneous CSD; (b) application of THC (5 µM), JWH 133 (20 µM), or WIN 55,215-2 mesylate (5 µM, 60 min) before the first injection of KCl (CSD1); (c) KCl injection, induction of CSD (CSD1); (d) washout of THC or WIN 55,215-2 mesylate, with ASCF (60 min) before the second injection of KCl (CSD2).

***Drugs***

WIN 55,212-2 and JWH 133 were both purchased from Tocris. THC was purchased from Sigma. All drugs were dissolved in DMSO. The final concentration of DMSO was less than 0.5%. All solutions used in control periods contained the same concentration of DMSO. 

***Statistical*** ***analysis***

All data are given as mean ± SEM. The data were statistically analysed using the paired student t test or Mann-Whitney rank sum test. Multiple comparisons were performed by analysis of variance test (ANOVA) for repeated measures followed by a Holm-Sidak’s test. Significance was established when the probability values were less than 0.05. The investigations were approved by the local ethics committee (Tierversuchsgenehmigung, Bezirksregierung Münster, Deutschland, AZ: 50.0835.1.0, G79/2002).

## Results

***The effect of THC on CSD***

Focal application of KCl induced negative DC deflections followed by positive waves (amplitude of 14.8 ± 1.7 mV; duration of 103 ±5 sec). Negative DC-fluctuations were sometimes preceded by small positive waves. These CSD waves propagated opposite to the direction of the ACSF flow at propagation velocity of 3.3 ± 0.1 mm / min. 

**Figure 1:**
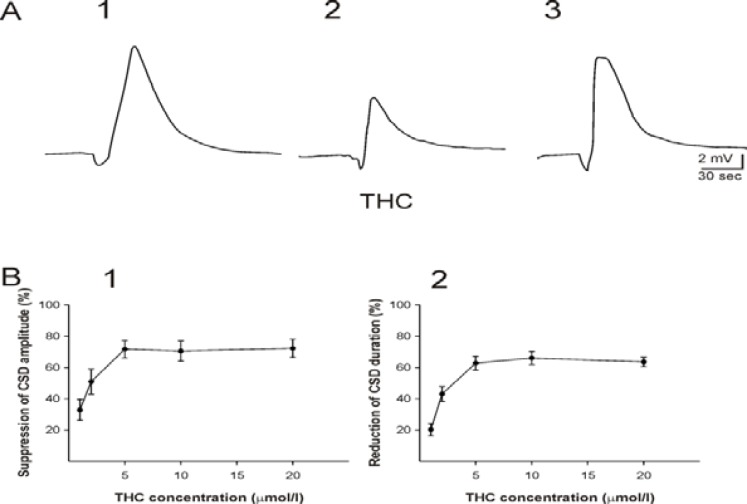
Effects of Delta9-tetrahydrocannabinol (THC) on cortical spreading depression (CSD) in the somatosensory neocortical tissues of rats. A: Recording of DC potentials in the third layer of a neocortical slice before (A1), during (A2), and after (A3) application of THC (5 µM). B: The relationship between THC concentrations and suppression of the amplitude (B1) and the duration (B2) of CSD. CSD was elicited by KCl microinjection. The curve indicates the plot of percentage reduction of CSD amplitude (B1) or duration (B2) vs. THC concentrations (n = 6 for each concentration). THC dose-dependently suppressed the amplitude and the duration of CSD (ANOVA, *P* ≤ 0.001). The percentage of CSD amplitude and duration reduction was measured by division of the amplitude and the duration of CSD induced after application of THC to the amplitude of SD elicited before superfusion of the substance. Values represent mean±SEM

The effect of five different concentrations of THC (0.1, 1, 2.5, 5, 10 µM; n = 6 for each concentration) was tested on potassium-evoked CSD in neocortical tissues. The ratio between the second and the first DC potential waves (CSD2/CSD1) was calculated in control slices and slices treated with THC. THC application at concentration of 0.1 µM did not significantly change different characteristic features of CSD, i.e. amplitude, duration, and propagation velocity. THC at higher concentrations (1-10 µM) dose-dependently reduced the amplitude and the duration of negative depolarisation potential shifts occurring after the second KCl application (Figure 1 A and B; ANOVA, *P* ≤ 0.001). THC at different concentrations decreased the amplitude and duration of CSD between 33±7 to 72±6 % and between 20±4 to 63±3 % of the initial levels, respectively. THC did not change the velocity of negative DC potential propagation at concentrations of 1-5 µM (3.2± 0.2 mm / min; *P* = 0.3). However, THC at 10 µM significantly decreased the velocity of DC-deflection propagation to 3±0.1 mm/min (t test, *P*≤ 0.006). After wash-out of THC, the amplitude, the duration, and the velocity of CSD propagation (CSD3) returned close to the initial levels (CSD1; Figure 1 A). 

THC (10 µM) was added to the bath medium sixty minutes before induction of the first CSD (amplitude of 10.4±1 mV; duration of 89±3 sec; the second protocol). Omission of THC from the bath medium significantly increased the amplitude of CSD to 13.7±1 mV (t test, *P* ≤ 0.006) and the duration to 109±2 sec (t test, *P* ≤ 0.003).

***The effect of CB (1)-agonist WIN 55,212-2 on CSD***

**Figure 2:**
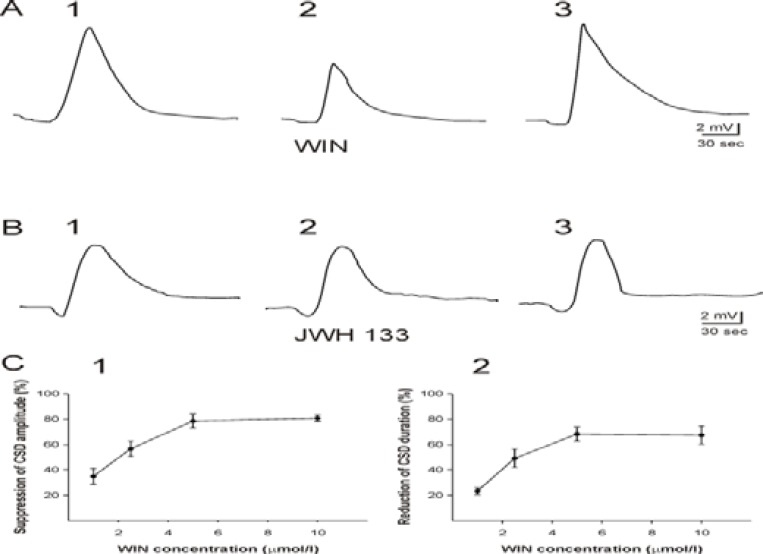
Effects of CB (1)-agonist WIN 55,212-2 and CB (2)-agonist JWH 133 on cortical spreading depression (CSD) in the somatosensory neocortical tissues of rats. A: Recording of DC potential shifts in the third layer of a neocortical slice before (A1), during (A2), and after (A3) application of WIN 55,212-2 (5 µM). WIN 55,212-2 significantly suppressed the amplitude and duration of CSD (t-test). B: Recording of negative DC-fluctuations before (B1), during (B2), and after (B3) application of JWH 133 (10 µM) in the third layer of a neocortical slice. There were no statistical changes in CSD characteristic features by JWH 133. SD was elicited by KCl microinjection. C: The curve indicates the plot of percentage decreases of CSD amplitude (C1) and duration (C2) vs. WIN 55,212-2 concentrations (n = 6 for each concentration). WIN 55,212-2 dose-dependently suppressed the amplitude (C1) and the duration of CSD (ANOVA, *P* ≤ 0.001). The percentage of CSD amplitude and duration reduction was measured by division of the amplitude and the duration of CSD induced after application of WIN 55,212-2 to the amplitude of SD elicited before superfusion of the substance. Values represent mean±SEM

WIN 55,212-2 at 0.1 µM did not affect CSD (n= 6). However, WIN 55,212-2 at concentrations of 1-10 µM dose-dependently decreased the amplitude of negative DC potentials which occurred after the second KCl application (CSD2, n=24, Figure 2 A; ANOVA, *P* ≤ 0.001). Application of WIN 55,212-2 for sixty min reduced the CSD amplitude to 33±4 % of the baseline level (CSD2/CSD1 ratio). WIN 55,212-2 at these concentrations also significantly and dose-dependently decreased the mean duration of CSD to 52±4 % of the initial value (ANOVA). WIN 55,212-2 only at concentrations of 5 and 10 µM significantly and reversibly decreased the velocity of the DC-wave propagation (2.7±1 mm / min; t test, *P* ≤ 0.001, Figure 2 A and C). After washout of the compound, the amplitude of the deflection of DC potentials (CSD3) returned close to the initial levels (CSD1; Figure 2 A). 

WIN 55,212-2 at concentration of 5 µM was added to ACSF sixty min before induction of CSD1 (n= 6, amplitude of 12.7±1 mV; duration of 70±4 sec). Omission of WIN 55,212-2 from the bath solution increased the characteristic features of the second CSD (amplitude of 19.2±1 mV; duration of 100±5 sec; t test, *P* ≤ 0.001). Application of DMSO at concentrations used to dissolve WIN 55,212-2 did not change the characteristic features of CSD (n= 8). 

***The effect of CB (2)-agonist JWH 133 on CSD***

Sixty min application of JWH 133 at concentrations of 1-20 µM after induction of CSD1 had no significant effects on the amplitude, the duration, and the velocity of propagation of the second CSD (n= 30, Figure 2 B). Furthermore, addition of JWH 133 (20 µM) to the bath medium before induction of the first CSD (the second protocol) also did not change the characteristic features of the first CSD comparing to the CSD elicited after sixty min wash-out of the substance (n= 6).

***The effect of WIN 55,212-2 on fEPSP and LTP***

**Figure 3.**
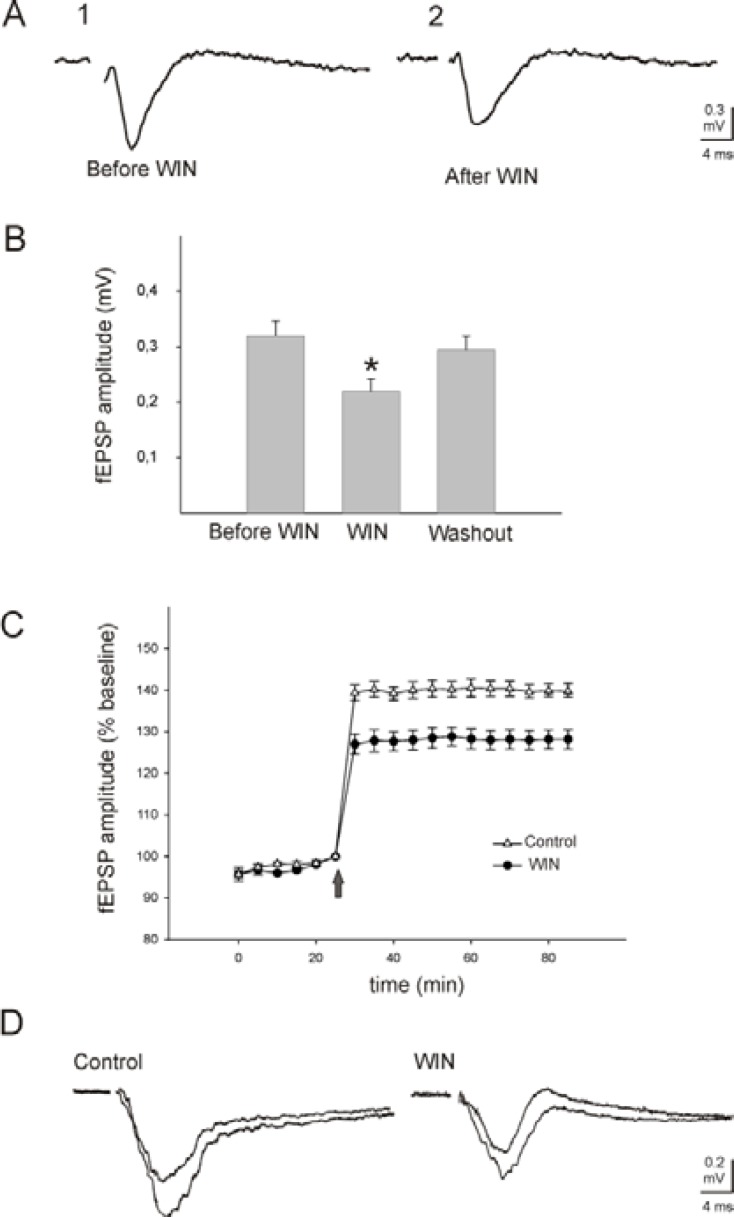
Effects of WIN 55,212-2 (WIN) on the evoked field excitatory post-synaptic potentials (fEPSP) and long-term potentiation (LTP) in the somatosensory neocortical tissues of rats. A: Recording of fEPSP in the third layer of a neocortical slice elicited by stimulation of white substance before (A1) and after (A2) application of WIN (5 μM). B: Group of bars represents the mean ± SEM of the amplitude of fEPSP before (first bar), during (middle bar) and after (third bar) application of WIN. C: Tetanic stimulation (ten trains of four pulses, pulse duration 0.1 msec; interpulse interval 50 msec) produces a rapid and stable potentiation in the amplitude of the fEPSP, calculated as a percentage of baseline mean response amplitude. Solid circles and open triangles show the evoked fEPSP after application of WIN (5 µM) and control, respectively. Arrow shows the time of tetanic stimulation, 60 min after application of WIN (5 µM) and artificial cerebrospinal fluid (ACSF, control). Application of WIN significantly inhibited LTP of the evoked field potentials ((Mann-Whitney rank sum test,* P*= 0.028), calculated as a percentage of baseline mean response amplitude. D: Representative examples of the evoked field potentials before and after tetanic stimulation in WIN and ACSF (control) affected slices. * indicates *P*= 0.028

The amplitude of the evoked fEPSP in the third layer of neocortical tissue by stimulation of white substance (with mean amplitude of 0.32 ± 0.03 mV) decreased within 5 min after addition of WIN 55,212-2 (5 μM; n= 10) to the superfusate. After 1 hr washing of the neocortical slices with WIN 55,212-2, the amplitude of the fEPSP significantly decreased to 31±0.2 % (Mann-Whitney rank sum test,* P*= 0.028) of the initial values (Figure 3 A and B). The suppressive effect of WIN 55,212-2 on the amplitude of the fEPSP was reversible. After wash-out of the compound, the amplitude of the fEPSP recovered nearly to the baseline level within 15 min (Figure 3 A and B).

A conditioning tetanic stimulation was delivered to the white substance of neocortical slices followed by pulses with stimulation parameters identical to control values. The evoked fEPSP was stable for at least 30 min before application of tetanic stimulation (less than 10% variation; Figure 3 C). Administration of tetanic stimulation produced a rapid and stable enhancement of the amplitude of the fEPSP in all tested preparations (n= 6, 140±1.7% control; Figure 3 C and D). The potentiation rose within 1–2 min and stabilized within 5 min after the train of stimulations. Application of WIN 55,212-2 (5 µM; n= 10) sixty min before tetanic stimulation significantly suppressed LTP induction in all tested slices (127±2.5% baseline, Mann-Whitney rank sum test, *P*≤ 0.001, Figure 3 C and D). LTP lasted as long as the fEPSP were recorded (at least for 90 min).

## Discussion

The present data revealed a dose dependent suppression of CSD by application of THC, the main active compound in cannabis. In addition, WIN 55,212-2, a CB1 receptor agonist, in contrast to JWH 133, a CB2 receptor agonist, also suppressed the amplitude, the duration, and the propagation velocity of CSD. These findings point to the involvement of CB1 receptors in generation and propagation of CSD. 

Two well-characterized cannabinoid receptors, CB1 and CB2, mediate the effects of cannabis in mammalian brain. CB1 cannabinoid receptors appear to mediate most of the psychoactive effects of THC and related compounds. This G protein-coupled receptor particularly expressed in cortex, hippocampus, amygdala, basal ganglia outflow tracts, and cerebellum, a distribution that corresponds to the most prominent behavioral effects of cannabis. CB2 cannabinoid receptors are also widely distributed in the mammalian brain. The multifocal expression of CB2 immunoreactivity in glial and neuronal patterns in a number of brain regions suggests the involvement of these receptors in depression and drug abuse (23). 

A broad functional expression of CB1 receptors in both GABAergic and glutamatergic neurons of the neocortex was reported (24). It was shown that the activation of presynaptic CB1 receptors decreases GABAergic synaptic inhibition (25, 26) and thus, may increase neuronal excitation by disinhibition. However, in the present study, we observed an inhibitory action on CSD by the activation of CB1 receptor. Several other studies also point to the inhibitory actions of CB1 receptors on neuronal activities. A lowered neuronal network excitability has been observed in rat neocortical slices in which the activation of CB1 receptors reduces the intensity and the spatial spread of the intrinsic optical signal and prolonged its kinetics (27). A decrease of neuronal excitation by application of CB1 receptor activation was also reported in the amygdale (28). It was demonstrated that THC or CB1 receptor agonist completely eliminates recurrent epileptic activity in a rat pilocarpine model of epilepsy (29). Endocannabinoid mobilization via presynaptic CB1 receptor dampening activity of the primary cortical output of neocortical neurons (30) and administration of THC decreases sensory-evoked cortical responses in anesthetized animals (31, 32). Conversely, disruption of endocannabinoid signalling by blocking of the CB1 receptors enhances whisker-evoked hyperemic responses in somatosensory cortex (33). The inhibitory effects of cannabinoid observed in the present study as well as other investigations are probably due to a decreased glutamatergic transmission, strong enough to override the disinhibitory effect on GABAergic transmission. 

Activation of CB1 receptors inhibits glutamatergic synaptic transmission. Both endogenous and synthetic cannabinoid receptor agonists activate potently and stereoselectively a presynaptic CB1 receptor that inhibits the release of glutamate via an inhibitory G-protein in cultured hippocampal pyramidal neurons (34-36). Hampson *et al* (36) have described an inhibitory modulation of the N-*Methyl*-D-*aspartate* (NMDA)-elicited signals, which is mediated by CB1 receptors in cortical and cerebellar cortices. In cerebellar granule neurons, cannabinoids modulate NMDA-mediated signals by interfering with calcium release from IP3-gated stores (37). The exposure during pregnancy to the CB1 receptor agonist causes impairment in neocortical glutamatergic neurotransmission and NMDA receptor functions in offspring (38). Activation of CB1 receptors inhibits the NMDA- and kainate-stimulated noradrenaline release in guinea-pig hippocampus as well as the NMDA-stimulated dopamine release in rat striatum (39) and blocks the neurotoxicity of NMDA in cultured rat hippocampal neurons (40). The original hypothesis regarding mechanism of initiation and propagation of CSD pointed to the crucial role of glutamatergic transmission (41). Indeed, activation of NMDA receptors is critical for generation and propagation of CSD in different neuronal tissues. It has been shown that the triggering of CSD requires activation of the NMDA subtype of glutamate receptors in human neocortical tissues (42), in rat cerebral cortex (43), and in chick retina (44). Blocking of NR2B-containing NMDA receptors also dose-responsively suppressed the CSD amplitude in rat neocortical tissues (45). In the presence of ifenprodil, an NR2B receptor subunit-selective NMDA receptor antagonist, the occurrence of CSD was abolished (46). 

However, other mechanisms of action such as modulation of NO, CGRP, or cyclooxygenase and lipoxygenase pathways may also contribute to the suppressive effect of cannabinoids on CSD. Anandamide, the endogenous ligand of the cannabinoid CB1 and CB2 receptors, was able to inhibit significantly neurogenic dural vasodilation, CGRP-, and NO-induced dural vessel dilation found in the rat intravital microscopy model of trigeminovascular activation (8). There is considerable evidence indicating NO and CGRP as key coupling compounds linking CSD changes in cerebral blood flow and metabolism (42). In addition, NO also plays a role in initiation and propagating of CSD. Local inhibition of NO synthesis with 7-nitroindazole, a selective neuronal NO synthase isoform, dose-dependently reduced the intensity of KCl induced SD in rats (47). The inhibitory effects of THC and other endocannabinoids on cyclooxygenase and lipoxygenase pathways such as their effects on phospholipase A_2_ and arachidonate metabolism (48) may also mediate their pharmacological actions on CSD. CSD induces a strong COX-2 mRNA expression in neocortex, which is regulated by NMDA receptor-stimulated phospholipase A_2_ (49).

In the present experiments, neocortical slices affected by the activation of CB1 receptors exhibited a pronounced and persisting inhibition of LTP. It has been known that synaptic plasticity and LTP depend on the availability of NMDA subtype glutamate receptors (50). Several investigations conducted on hippocampal tissues have indeed shown that cannabinoids act at CB1 receptors prevent induction of LTP (51, 52). Disrupting CB1 receptor-mediated neurotransmission at the genome level produces mutant mice with an enhanced capacity to strengthen synaptic connections (53, 54), suggesting that endocannabinoids restrict the potentiation process. CSD induces an LTP-like effect in rat neocortical slices (55) and enhances LTP induction in human neocortical tissues (56). A facilitatory effect of CSD on induction of LTP has been reported (57, 58). Enhancement of LTP induction and facilitation of CSD occurrence was observed by application of female hormones in rat somatosensory neocortical tissues (59). Modulation of LTP was also observed isolated from the CSD propagation site in hippocampal tissues (60). It has been shown that enhancement of synaptic strength was accompanied with cellular hyperexcitability (61, 62). The inhibition of LTP after CB1 agonist application could be due to blockade of NMDA receptors/channels at the synaptic site, an effect which may be also responsible for its suppressive effect on CSD. 

It has been shown that neurons with A-fiber and C-fiber input in the trigeminocervical complex with input from the ophthalmic division of the trigeminal nerve were inhibited by activation of the cannabinoid CB1 receptors (9). Moskowitz *et al* (17) suggested that CSD activates trigeminal afferents, thus causing the pain and the cascade of events recognized as migraine. Although this theory was challenged by some studies (63) and is still a matter of debate (15), still there exists a link between the visual aura and pain by showing that CSD triggers trigeminal afferents in rats. This link demonstrated that CSD induces a delayed blood flow increase within the pial vessels and middle meningeal artery, causing protein leakage in dura mater, and activating the ipsilateral trigeminal nucleus caudalis (18). Furthermore, intracellular recordings of the neurons in the dorsal horn of cervical spinal cord segment, ipsilateral to the hemisphere in which CSD was evoked, showed a transient suppression of spontaneous burst discharges, followed by a significant enhancement of the neuronal activity. This suggested sensitization and activation of the neurons responsible for processing sensory information in the trigeminocervical complex by CSD (19). Activation of CB1 receptors may alleviate migraine pain by inhibition of CSD and its consequent trigeminal neuronal activation. Several anti-migraine drugs, such as propranolol, sumatriptan, methysergide, paracetamol, acetylsalicyclic acid, and dihydroergotamine, suppressed different characteristic features of CSD in various animal models, both *in vivo* and *in vitro* (42). 

There may be some *limitations* that need to be acknowledged and addressed regarding the use of cannabinoids in treatment of SD-related disorders such as migraine headache. The first *limitation* concerns the hallocinogenic properties of cannabinoids. This side-effect should be taken into consideration in further development of new cannabinoid derivatives as new drug (1). In addition, cannabinoid CB1 agonist inhibited induction of LTP in our study. Changing of synaptic plasticity by activation of CB1 receptors may affect signal processing as well as learning and memory in different regions of the brain.

Suppression of CSD by modulation of CB1 receptors may point to the potential therapeutic effects of cannabinoids in migraine with aura. More experimental and clinical researches are needed before we know whether cannabinoids may really be helpful in treating migraine pain.

## Conclusions

Suppression of CSD by activation of CB1 receptors suggests the potential therapeutic effects of cannabinoids in migraine with aura as well as other CSD-related disorders. More research is needed before we know whether cannabinoids may be helpful in treating migraine pain.
